# Selective Cellular Uptake and Druggability Efficacy through Functionalized Chitosan-Conjugated Polyamidoamine (PAMAM) Dendrimers

**DOI:** 10.3390/s24154853

**Published:** 2024-07-26

**Authors:** Ye Hu, Jian Chen, Wenyan Hu

**Affiliations:** 1 Nanjing Institute for Food and Drug Control, Nanjing 211198, China; 2 School of Chemistry and Chemical Engineering, Hunan University of Science and Technology, Xiangtan 411201, China

**Keywords:** chitosan, PAMAM, drug delivery, cellular internalization, druggability

## Abstract

Nanotechnology has ushered in significant advancements in drug design, revolutionizing the prevention, diagnosis, and treatment of various diseases. The strategic utilization of nanotechnology to enhance drug loading, delivery, and release has garnered increasing attention, leveraging the enhanced physical and chemical properties offered by these systems. Polyamidoamine (PAMAM) dendrimers have been pivotal in drug delivery, yet there is room for further enhancement. In this study, we conjugated PAMAM dendrimers with chitosan (CS) to augment cellular internalization in tumor cells. Specifically, doxorubicin (DOX) was initially loaded into PAMAM dendrimers to form DOX-loaded PAMAM (DOX@PAMAM) complexes via intermolecular forces. Subsequently, CS was linked onto the DOX-loaded PAMAM dendrimers to yield CS-conjugated PAMAM loaded with DOX (DOX@CS@PAMAM) through glutaraldehyde crosslinking via the Schiff base reaction. The resultant DOX@CS@PAMAM complexes were comprehensively characterized using Fourier-transform infrared (FTIR) spectroscopy, transmission electron microscopy (TEM), and dynamic light scattering (DLS). Notably, while the drug release profile of DOX@CS@PAMAM in acidic environments was inferior to that of DOX@PAMAM, DOX@CS@PAMAM demonstrated effective acid-responsive drug release, with a cumulative release of 70% within 25 h attributed to the imine linkage. Most importantly, DOX@CS@PAMAM exhibited significant selective cellular internalization rates and antitumor efficacy compared to DOX@PAMAM, as validated through cell viability assays, fluorescence imaging, and flow cytometry analysis. In summary, DOX@CS@PAMAM demonstrated superior antitumor effects compared to unconjugated PAMAM dendrimers, thereby broadening the scope of dendrimer-based nanomedicines with enhanced therapeutic efficacy and promising applications in cancer therapy.

## 1. Introduction

PAMAM dendrimers, with a particular focus on their recent advancements in drug delivery, gene transfection, and contrast enhancement for magnetic resonance imaging (MRI), offer innovative avenues in clinical and pharmaceutical applications [1,2]. Their unique structure, characterized by a well-ordered and highly branched three-dimensional architecture stemming from the core and interior repeating units, presents promising opportunities for small and large molecule drug delivery and release. The choice of core and repeating units profoundly influences the properties of PAMAM dendrimers, including their hydrophilicity, mechanical strength, and size characteristics. Their synthetic versatility and structural homogeneity endow PAMAM dendrimers with diverse nano-scale dimensional structures [3]. Notably, their extensive branching and hydrophobic internal voids make them excellent candidates for the delivery of hydrophobic drugs. For instance, Wang et al. devised label-free fluorescent PAMAM dendrimers for traceable and controlled drug delivery, showcasing accelerated drug release kinetics within acidic environments [4]. The hydrophobic cavities within PAMAM dendrimers offer natural advantages for encapsulating poorly water-soluble drugs, yet challenges persist [5,6]. Despite their efficacy as drug carriers, unresolved issues remain, reflecting the dual nature of PAMAM dendrimers [7]. Concerns regarding drug delivery processes, stability, and cellular uptake necessitate further investigation, particularly regarding their utilization as improved carriers for tumor therapy. Challenges such as unsatisfactory drug release kinetics, rapid clearance by the reticuloendothelial system, intrinsic cytotoxicity, and limited hydrophilicity impede their practical applications. Addressing these issues is crucial to unlock the full potential of PAMAM dendrimers in anti-tumor drug delivery. Efforts to enhance their biodistribution, cellular uptake, and stability hold promise for overcoming current limitations and advancing their utility in clinical settings [8].

In general, potential research avenues include the utilization of zwitterion-grafted PAMAM dendrimers [9], pegylated PAMAM dendrimers [10], and phosphorylated serine-modified PAMAM dendrimers [11]. Leveraging PAMAM dendrimers can significantly enhance the bioavailability of drugs due to their structured order, expanded contact surface, and heightened mechanical and chemical stability [12,13]. The modular synthetic route of PAMAM dendrimers, characterized by a core with repeated branches, facilitates facile access. The generations of branches, determined by the number emanating from the core, feature numerous active amine groups on the surface. This enables easy modification with various ligand groups on the periphery, allowing for adjustments in their physical, chemical, and in vivo properties. The incorporation of chitosan (CS) with PAMAM dendrimers represents a promising strategy for addressing existing challenges. The abundance of surface functional groups on PAMAM dendrimers, amenable to chemical conjugation, underscores the advantages of dendrimer modification. Techniques such as orthogonal and ‘click’ chemistries offer straightforward synthetic routes for coating PAMAM dendrimers with CS, further enhancing their functionality and applicability [14,15].

CS is a linear polysaccharide comprising β-1,4-glycosidic linkages between D-glucosamine and *N*-acetyl-d-glucosamine units, rendering it a vital natural polysaccharide material [16]. Notable for its excellent biocompatibility, biodegradability, non-toxicity, antimicrobial properties, porosity, and mucoadhesivity, CS stands out in biomedical applications. The abundant hydrophilic groups, including hydroxy, amino, and carboxyl groups on CS glucose units, confer remarkable water absorption capabilities, facilitating the transformation of CS solutions into hydrogels [17]. This property enhances moisture absorption and promotes cell adhesion, as demonstrated by Li et al.’s development of core-shell chitosan microspheres with antimicrobial and vascularization functions for enhancing skin wound healing [18] and Cheng et al.’s exploration of nano-hydroxyapatite/chitosan composite microspheres to promote osteogenesis and angiogenesis for bone regeneration [19]. Additionally, Rajan et al. demonstrated the potential of poly-carboxylic acid-functionalized chitosan nanocarriers for controlled and targeted anti-cancer drug delivery [20]. CS plays a pivotal role in combating infections, promoting wound healing, tissue regeneration, and anticancer effects, particularly in enhancing cell adhesion. CS conjugation offers a solution to overcome limitations associated with PAMAM dendrimers, such as instability in aqueous environments, immunogenicity, systemic cytotoxicity, and hemolytic toxicity, while also enhancing the solubilization of hydrophobic drugs and facilitating drug delivery and tumor targeting [21]. Furthermore, CS aids in traversing biological barriers, enhancing the ease of drug transport. The advent of CS conjugation has sparked interest in leveraging CS as a versatile platform for drug carrier modification. Numerous studies have focused on surface tailoring of nanoparticles with CS to enhance biocompatibility and cell adhesion capabilities, thereby broadening the potential applications of CS-based drug delivery systems [22,23,24].

**Figure 1:**
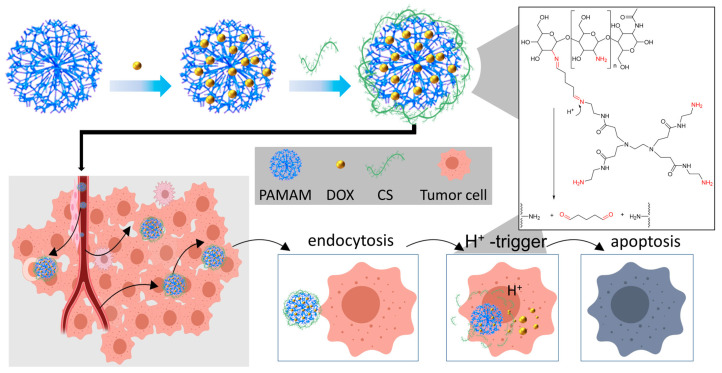
The assembly, cellular internalization, and drug release behavior of DOX@CS@PAMAM.

In this study, we investigated functional CS-conjugated PAMAM dendrimers for enhanced antitumor effects. A comparison between the efficacy of functional CS-conjugated PAMAM dendrimers and unconjugated PAMAM dendrimers was conducted in vitro. Initially, PAMAM G3.0 was selected for modification due to its optimal size and excellent entrapment efficiency, minimizing drug leakage. The hydrophobic drug DOX was encapsulated within the branched three-dimensional structure of PAMAM dendrimers using a methanol solution. Subsequently, water-soluble CS was conjugated with DOX-loaded PAMAM dendrimers via the classical glutaraldehyde cross-linking method to impart acid responsiveness (Figure 1). The physical and chemical properties of the resulting DOX@CS@PAMAM, including the structural validation and size, were thoroughly characterized. In vitro experiments validated the expected responsive release profiles. Most notably, the improved antitumor effect of DOX@CS@PAMAM was confirmed through assessments of cell viability, cell imaging, and flow cytometry analysis. These findings underscore the potential of functional CS-conjugated PAMAM dendrimers as promising candidates for enhancing antitumor efficacy.

## 2. Experimental Section

### 2.1. Materials

PAMAM dendrimer (G3) was procured from Weihai CY Dendrimer Technology Co., Ltd. (Weihai, China), while chitosan, glutaraldehyde (50% aqueous solution), NaH_2_PO_4_, Na_2_HPO_4_, NaCl, and doxorubicin were obtained from Tansoole (Shanghai, China). All reagents were utilized without further purification, and solvents were purified following standard procedures prior to usage.

### 2.2. Preparation of DOX-Loaded PAMAM

A total of 20 mg of DOX was dissolved in 200 μL of MeOH in an EP tube, followed by the addition of 20 mg of PAMAM to the same tube. The solution was vortexed at room temperature for 2 h. Subsequently, the mixture was injected into 50 mL of deionized water using a micro syringe and stirred for an additional 2 h at room temperature. The resulting solution was then transferred into a dialysis bag (MWCO = 3000) to remove any unloaded DOX. Dialysis was carried out for a minimum of 72 h in darkness. Finally, the solution was subjected to freeze-drying to remove water, yielding a freeze-dried powder (DOX@PAMAM) for subsequent modifications.

### 2.3. Drug Loading Content of DOX-Loaded PAMAM

A total of 10 mg of the freeze-dried powder was dissolved in 500 μL of PBS solution (pH = 7.4). The drug loading content of DOX-loaded PAMAM was determined using the Thermo Fisher Varioskan LUX (Thermo Scientific™, Waltham, MA, USA) for multifunction microplate reader. A calibration curve was established with various DOX concentrations (y = 0.044449x + 0.021, R^2^ = 0.9925). The drug loading content was calculated using the following equation: drug loading content = (weight of DOX/weight of DOX-loaded PAMAM) × 100%. The drug loading content of DOX@PAMAM was analyzed in triplicate, yielding values of 36.4%, 37.1%, and 39%. The average drug loading content was reported as 37.5 ± 1.3%. The drug encapsulation efficiency was calculated using the following equation: drug encapsulation efficiency = (weight of DOX − weight of non-encapsulated DOX)/weight of DOX × 100%. The drug encapsulation efficiency of DOX@PAMAM was also analyzed in triplicate, yielding values of 72.8%, 74.2%, and 78%. The average drug encapsulation efficiency was reported as 75 ± 2.6%. All samples were analyzed in triplicate, and the averages were reported.

### 2.4. Preparation of CS-Conjugated PA MAM Dendrimer Loaded with DOX

A total of 10 mg of DOX-loaded PAMAM powder was dissolved in 200 μL of PBS (pH = 7.4) in an EP tube. Subsequently, 10 mg of CS was added to the solution and vortexed at room temperature for 30 min. Following this, 50 μL of glutaraldehyde was added to the solution and further vortexed at room temperature overnight. The solvent of the resulting DOX@CS@PAMAM solution was then transferred into a dialysis bag (MWCO = 10,000) to eliminate any unreacted CS and glutaraldehyde. Finally, the obtained DOX@CS@PAMAM was subjected to freeze-drying to yield the final product.

### 2.5. Drug Loading Content of CS-Conjugated PAMAM Dendrimer Loaded with DOX

The procedure parallels the drug-loading content determination for DOX-loaded PAMAM. The obtained drug loading content of DOX@CS@PAMAM was 30%. The drug loading content of DOX@PAMAM was analyzed in triplicate, yielding values of 29.1%, 29.9%, and 31%. The average drug loading content was reported as 30 ± 1%. The drug encapsulation efficiency of DOX@PAMAM was also analyzed in triplicate, yielding values of 58.1%, 59.8%, and 62%. The average drug encapsulation efficiency was reported as 60 ± 2%. All samples were analyzed in triplicate, and the averages were reported.

### 2.6. Characterization

FTIR data were collected using the Thermo Scientific Nicolet iS50 (Thermo Scientific™, Waltham, MA, USA) with 32 scans at a resolution of 4 cm^−1^. For sample preparation, 1 mg of the samples was mixed with 200 mg of spectrally pure KBr in a grinding bowl to form KBr pellet mixtures. TEM analysis was performed using the Tecnai G2 F20 S-TWIN (FEI Company, Hillsborough, OR, USA) operating at 200 kV. Drops of the samples were deposited onto copper grids and allowed to air dry naturally to remove water. DLS experiments were conducted using a Malvern Zetasizer Pro. The powder samples were dissolved in PBS (pH = 7.4) solution, diluted to 1 mg/mL, and then filtered through a 0.45 μm Millipore filter prior to analysis. All samples were analyzed in triplicate, and the averages were reported.

### 2.7. In Vitro Drug Release

Drug release experiments were conducted using both an acidic PBS solution (pH = 4.5) to simulate the acidic tumor environment and a neutral PBS solution (pH = 7.4) to represent the normal tissue environment. Briefly, 10 mg of either DOX@PAMAM powder or DOX@CS@PAMAM was dissolved in 1 mL of PBS solution and transferred into dialysis tubes with a molecular weight cut-off (MWCO) of 3000. These dialysis tubes were then immersed in larger tubes containing 5 mL of either acidic PBS solution (pH = 4.5), neutral PBS solution (pH = 7.4), or alkaline PBS solution (pH = 9). At predetermined time intervals, samples of the external dialysis solution were withdrawn and analyzed using a multifunction microplate reader. Subsequently, the tested dialysis tubes were returned to the larger tubes to continue the sequential drug release process.

### 2.8. Cell Viability Test

SKOV-3 cells were cultured in DMEM (Gibco)(Thermo Scientific company, Waltham, MA, USA) supplemented with 10% FBS (Gibco) and 1% penicillin–streptomycin. The cells were maintained at 37 °C in a humidified atmosphere containing 5% CO_2_. Prior to the experiment, SKOV-3 cells were seeded into 96-well plates at a density of 1 × 10^4^ cells per well. After 24 h of incubation, the cells were treated with various concentrations of unconjugated PAMAM or CS@PAMAM for an additional 24 h. Cell viability was assessed using the CCK-8 reagent (Beyotime Biotechnology company, Shanghai, China). Specifically, 10 μL of the CCK-8 reagent was added to each well, followed by incubation for 2 h at 37 °C. The absorbance at 450 nm was measured using a multifunction microplate reader, and the percentage of cell viability was calculated relative to untreated control cells. For the pH-dependent cytotoxicity assay of DOX@PAMAM or DOX@CS@PAMAM, SKOV-3 cells were treated with various concentrations of DOX@PAMAM or DOX@CS@PAMAM in either acidulated culture medium or normal culture medium for 24 h. Subsequently, cell viability was assessed using the CCK-8 assay.

### 2.9. Fluorescence Imaging Analysis

SKOV-3 cells seeded in culture dishes were treated with free DOX, DOX@PAMAM, and DOX@CS@PAMAM dendrimers at a concentration equivalent to a DOX concentration of 72 ng/mL in either acidulated culture medium or normal culture medium. After 3 h, the cells were washed with PBS three times, fixed with 4% paraformaldehyde (Beyotime Biotechnology company, Shanghai, China), and stained with DAPI (Beyotime Biotechnology company, Shanghai, China) to label the nuclei. Images were acquired using an Olympus ix73 microscope. DOX fluorescence was excited using a 488 nm laser, and emission was collected from 550 to 620 nm. DAPI fluorescence was excited using a 340 nm laser, and emission was collected from 420 to 480 nm.

### 2.10. Flow Cytometry Analysis

SKOV-3 cells (1 × 10^5^ cells/mL) were seeded in six-well plates and allowed to incubate overnight. Following this, free DOX, DOX@PAMAM, and DOX@CS@PAMAM (each at an equivalent DOX concentration of 72 ng/mL) in either acidulated culture medium or normal culture medium were added to each well. The plates were then further incubated for 3 h. Cells without any treatment served as the negative control. Subsequently, the cells were trypsinized and washed with PBS buffer three times to remove any physically adsorbed samples from the cell surface. The uptake of samples containing DOX was measured using a flow cytometer (Beckman CytoFlex) (Beckman Coulter company, CA, USA), with excitation at 480 nm and emission collected at 585 nm. Six groups of blank SKOV-3 cells were, respectively, used as the intracellular DOX-negative gating control, and the intracellular DOX in SKOV-3 cells was analyzed and quantified using FlowJo software (V10.8.1).

## 3. Results and Discussion

**Figure 2:**
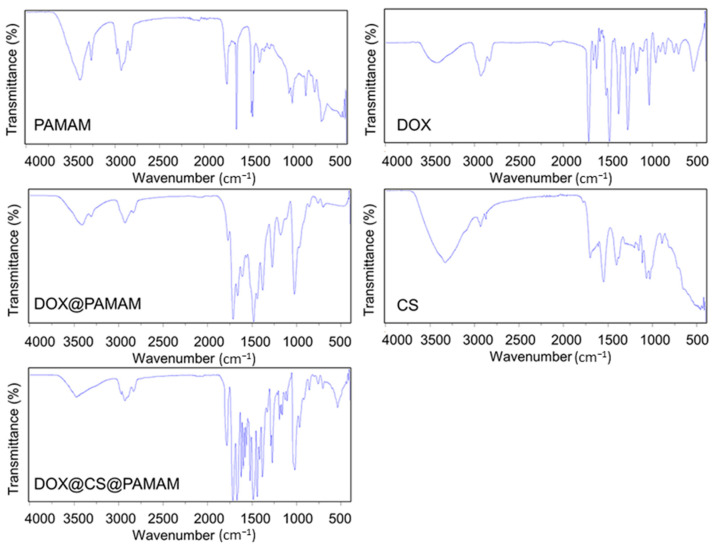
The FTIR spectra of PAMAM, free DOX, DOX@PAMAM, CS, and DOX@CS@PAMAM.

According to our assumptions, we designed a functional CS-conjugated PAMAM dendrimer to enhance the antitumor effect and address the shortcomings of existing PAMAM dendrimers. Thus, improving the physicochemical properties of PAMAM dendrimers, particularly their hydrophilicity and cell adhesion, holds significant importance. To confirm the successful preparation of this anticipated functional CS-conjugated PAMAM dendrimer, FTIR was employed to analyze the changes resulting from the conjugation process. As depicted in Figure 2, for the unmodified PAMAM dendrimer, characteristic absorption peaks were observed. The peak at 3300 cm^−1^ corresponded to secondary amides, while peaks at 3250 cm^−1^ indicated amino stretching vibrations. Peaks at 2950 cm^−1^ and 2890 cm^−1^ were attributed to asymmetric and symmetric stretching vibrations of methylene groups in PAMAM, respectively. The peak at 1740 cm^−1^ represented carbonyl stretching vibrations, and the peak at 1660 cm^−1^ denoted amide stretching vibrations. Additional peaks at 1120 cm^−1^ and 1022 cm^−1^ were attributed to primary and tertiary amine stretching vibrations, respectively [25]. These characteristic peaks confirmed the presence of NH2, CH2, and CONH groups in PAMAM, consistent with its theoretical structure. Upon loading doxorubicin (DOX) onto the pristine PAMAM, characteristic peaks of DOX emerged, including peaks at 3200–3500 cm^−1^ (OH and NH stretching vibrations), 2950 cm^−1^ (CH stretching in benzene ring), 1730 cm^−1^ (C=O stretching), 1600 cm^−1^ and 1500 cm^−1^ (C=C stretching in benzene ring), 1380 cm^−1^ (symmetric deformation vibration of OCH3), and 1000 cm^−1^ (CO bond stretching). For chitosan (CS), characteristic peaks were observed at 3300 cm^−1^ (hydroxy and amino stretching vibrations), 1660 cm^−1^ and 1560 cm^−1^ (C=O stretching and NH2 bending vibrations of amide), 1400 cm^−1^ (CH_3_ bending vibration of COCH_3_), and 1160 cm^−1^, 1070 cm^−1^, 1022 cm^−1^, and 890 cm^−1^ (skeletal vibrations of the pyranose unit). In the case of DOX@CS@PAMAM, interaction between PAMAM and CS led to variations in the FTIR spectra compared to individual components. Notably, a distinct peak at 1650 cm^−1^, assigned to the imine bond, indicated crosslinking between PAMAM and CS. Additionally, peaks at 2960–2850 cm^−1^ were attributed to CH stretching vibrations of glutaraldehyde, a crosslinker used in the conjugation process. Furthermore, common characteristic peaks of PAMAM and CS were observed in the range of 850 cm^−1^ to 1750 cm^−1^. These findings confirm the successful conjugation of CS onto DOX@PAMAM dendrimers.

**Figure 3:**
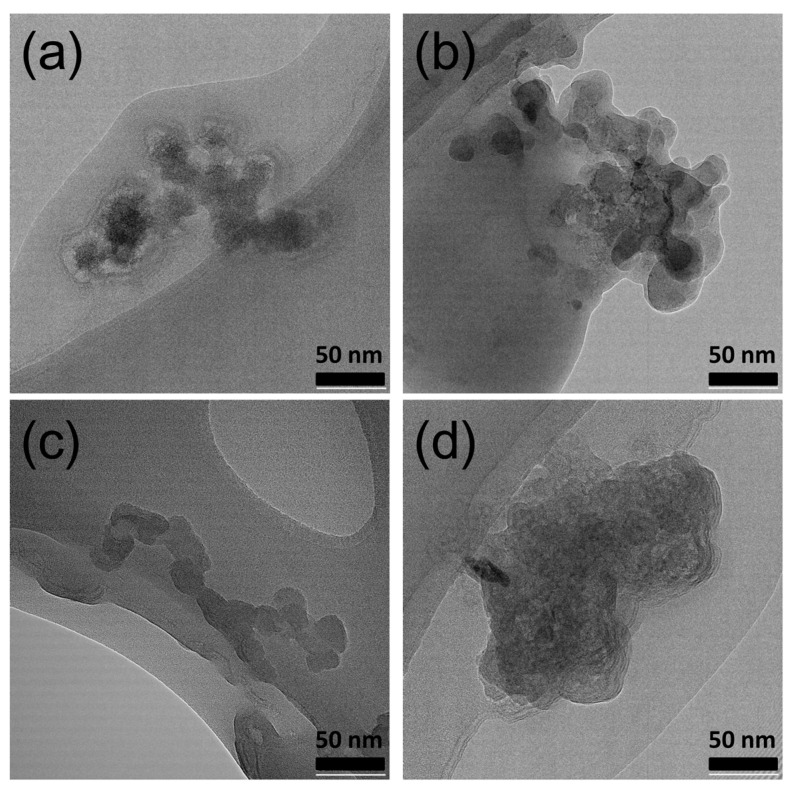
TEM images of PAMAM (**a**), DOX@PAMAM (**b**), CS (**c**), and DOX@CS@DOX (**d**). Scale bar: 50 nm.

While FTIR analysis confirmed the conjugation between PAMAM and CS, the underlying assembly processes governing the evolution of these materials during synthesis remain poorly understood. To address this gap, TEM was employed to investigate the morphological evolution process of CS-conjugated PAMAM dendrimers. This analysis aimed to elucidate the intrinsic correlation between structural changes in depth. As illustrated in Figure 3, individual PAMAM dendrimers exhibited a morphology closely resembling their actual size of approximately 50 nm. Subsequently, DOX was loaded onto the PAMAM dendrimers. The resulting DOX-loaded PAMAM dendrimers displayed a significantly increased size compared to individual PAMAM dendrimers, suggesting that the loaded DOX induced spatial expansion of the PAMAM dendrimers. Additionally, individual CS molecules were observed via TEM, revealing a long linear chain-like structure. However, after the modification process and conjugation with PAMAM dendrimers, the morphology of the materials transitioned from a branched three-dimensional structure to a spherical shape. These observations indicate that CS could be conveniently conjugated on the surface of PAMAM dendrimers, leading to structural alterations and the formation of spherical structures.

The TEM results provided significant insights into the hydration status of these materials during synthesis. Functionally, CS-conjugated PAMAM dendrimers are expected to be hydrated in practical applications. Therefore, observing the size distributions of functional CS-conjugated PAMAM dendrimers in an aqueous environment more accurately reflects their true state.

**Figure 4:**
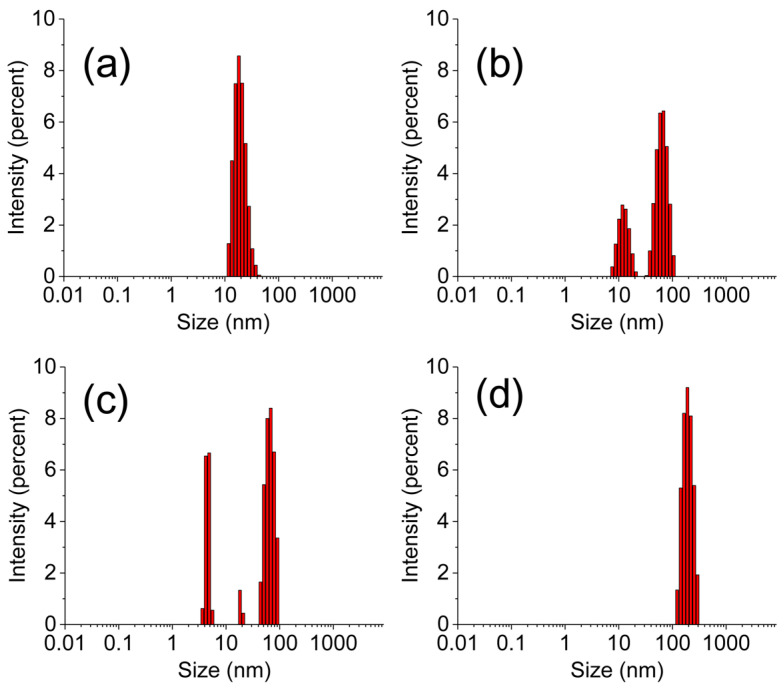
The DLS of PAMAM (**a**), DOX@PAMAM (**b**), CS (**c**), and DOX@CS@PAMAM (**d**).

The primary objective of this study was to prepare the CS-conjugated counterpart and assess its performance, particularly focusing on increased cellular internalization. For the unconjugated PAMAM dendrimer, the hydrated particle size was observed to be approximately 70 nm ± 8.7 nm with a narrow size distribution. The preparation process of commercial PAMAM has been fully refined, allowing for precise control over its size. As a result, the size distribution of pure PAMAM is narrow and exhibits a monomodal distribution. Upon loading with DOX, the hydrated particle exhibited a larger size compared to the individual one, attributable to the presence of loaded DOX, which enhances the hydrophobic interactions of PAMAM and causes agglomeration in an aqueous environment. Consequently, the DLS results showed a heterogeneous size distribution. Similarly, individual CS molecules displayed an average hydrated particle size of approximately 50 ± 5.6 nm. Due to its chain structure, CS disperses in an aqueous environment and does not form spherical structures like PAMAM. Consequently, the size distribution of CS is non-uniform. Notably, the functional CS-conjugated PAMAM dendrimer exhibited a particle size of approximately 200 ± 13.5 nm. This size reduction, compared to the unconjugated PAMAM dendrimer, was accompanied by a narrow size distribution (Figure 4). These findings suggest that CS conjugation enhances dispersion stability in aqueous environments, preventing agglomeration and coagulation. As a result, the size distribution of DOX@CS@PAMAM is more centralized and uniform. Moreover, CS contributes to superior hydrophilicity, which is advantageous for cellular internalization. This enhanced hydrophilicity, coupled with the optimized particle size and distribution, underscores the potential of functional CS-conjugated PAMAM dendrimers for improved cellular uptake and biomedical applications.

**Figure 5:**
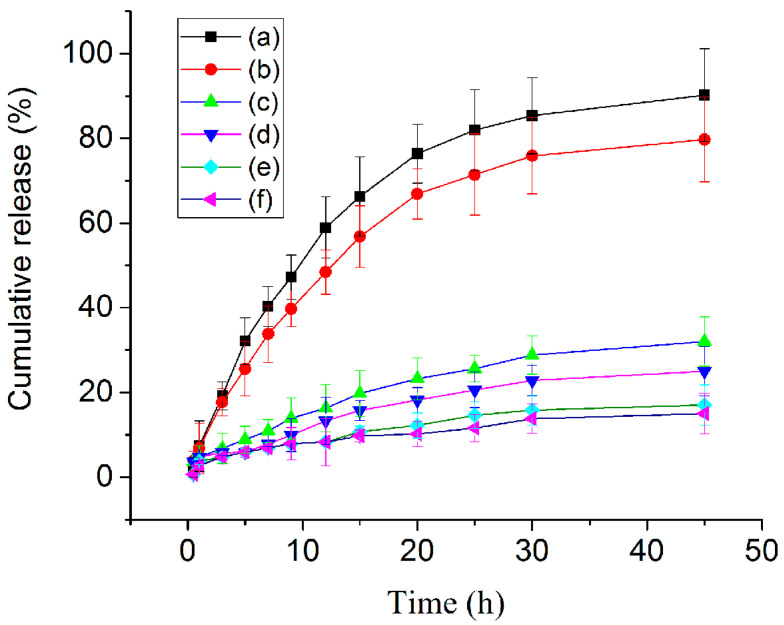
The drug release profiles of DOX@PAMAM in acidic PBS solution (pH = 4.5) (a), DOX@CS@PAMAM in acidic PBS solution (pH = 4.5) (b), DOX@PAMAM in neutral PBS solution (pH = 7.4) (c), DOX@CS@PAMAM in neutral PBS solution (pH = 7.4) (d), DOX@PAMAM in alkaline PBS solution (pH = 9) (e), and DOX@CS@PAMAM in alkaline PBS solution (pH = 9) (f).

Tumor tissues are known for their high metabolic activity, leading to the accumulation of acidic metabolic byproducts and the creation of an acidic microenvironment. Leveraging this characteristic, we developed a functional CS-conjugated PAMAM dendrimer using an acidity-sensitive imine linkage. The lysosomes of cancer cells exhibit lower pH levels (3.8–4.7) compared to those of normal cells (pH 4.5–6.0) [26]. To evaluate their potential for controlled drug release in tumor cells, we conducted drug release studies in a simulated tumor microenvironment with a pH of 4.5 (Figure 5). In this acidic environment, the accumulative release of DOX from the unconjugated PAMAM dendrimer reached 90% within 45 h, demonstrating efficient drug release. Even the functional CS-conjugated PAMAM dendrimer showed remarkable acid-responsive behavior, achieving a 75% accumulative release within the same timeframe. While the drug release profile of DOX@CS@PAMAM in acidic environments was inferior to that of DOX@PAMAM, several factors may account for this difference, including internal mass transfer resistance and intermolecular interaction forces introduced by CS. Correspondingly, the presence of CS encapsulation and imine crosslinking in the functional dendrimer led to a less smooth release of DOX. Interestingly, both the unconjugated and CS-conjugated PAMAM dendrimers exhibited slow DOX release in a normal simulated environment, indicating controlled release characteristics. Moreover, they demonstrated an even more limited and slower release of DOX in an alkaline simulated environment (pH 9.0). These results highlight the promising release behavior of the developed functional CS-conjugated PAMAM dendrimer in simulated environments, suggesting its potential for tumor intracellular drug delivery.

**Figure 6:**
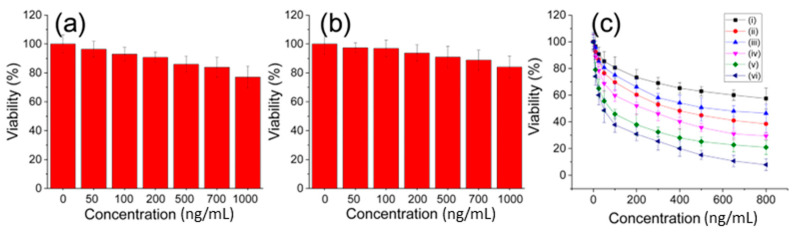
The biocompatibility of PAMAM, namely, unconjugated PAMAM (**a**), CS@PAMAM, namely, CS conjugated PAMAM (**b**) and the cytotoxicity (**c**) of DOX@PAMAM in a normal environment (i), DOX@CS@PAMAM in a normal environment (ii), DOX@PAMAM in an acidic environment (iii), DOX@CS@PAMAM in an acidic environment (iv), and free DOX in a normal environment (v) and in an acidic environment (vi).

The potential autotoxicity of the functional CS-conjugated PAMAM dendrimer needed to be assessed to determine its suitability for developing novel formulations. Hence, the cytotoxicity of the unloaded PAMAM dendrimer and the functional CS-conjugated PAMAM dendrimer was evaluated under the same conditions using the CCK-8 method (Figure 6a,b). Generally, both the PAMAM dendrimer and CS@PAMAM themselves exhibited low toxicity to the cultured cells, especially at relatively low concentrations. Even at concentrations of up to 1000 ng/mL, the cell viability remained above 80%. Therefore, the biocompatibility of the functional CS-conjugated PAMAM dendrimer was deemed acceptable. However, the focus lay on the DOX-loaded functional CS-conjugated PAMAM dendrimer for its anti-tumor effects, aiming to achieve improved performance surpassing the old formulation. In the acidic tumor environment, the cell culture medium was weakly acidified to simulate this acidic environment. In acidulated tumor cells, DOX@CS@PAMAM exhibited potent antitumor activity (Figure 6c, iv) with an IC50 of 240 ng/mL (equivalent to a DOX concentration of 72 ng/mL) due to its selective cellular internalization and acid-responsive drug release. Comparatively, the antitumor profile of the unconjugated PAMAM dendrimer was inferior to that of the functional CS-conjugated PAMAM dendrimer, limited by poor cellular internalization and hydrophilicity. In a simulated normal physiological environment, the DOX-loaded functional CS-conjugated PAMAM dendrimer demonstrated superior tumor cell killing effects compared to the unconjugated PAMAM dendrimer, indicating that CS conjugation using glutaraldehyde crosslinking favored controllable drug release and cellular internalization, resulting in enhanced tumor killing efficacy. To highlight the advantage of the CS-conjugated PAMAM dendrimer, free DOX was also used to treat tumor cells in both simulated acidic and normal environments (Figure 6c, v and vi). However, free DOX directly acted on tumor cells, exerting strong cytotoxicity and causing significant damage to tumor cells. This underscores the importance of the CS-conjugated PAMAM dendrimer in drug delivery and tumor site-directed release.

**Figure 7:**
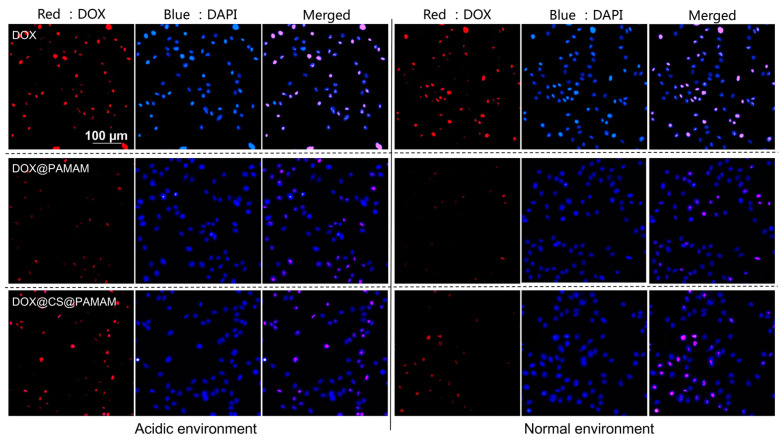
Fluorescence imaging profile of Skov 3 cells that were incubated with free DOX, DOX@PAMAM, and DOX@CS@PAMAM in an acidic environment and a normal environment.

Intracellular fluorescence imaging was employed to visualize the intracellular drug distribution, facilitating intuitive color changes and enabling the understanding of cellular uptake behavior of CS-conjugated PAMAM dendrimers. To validate the feasibility of this drug carrier in mimicking tumor acidic microenvironments and normal physiological environments, tumor cells were treated with acidulated culture medium using acidic PBS solution and unacidified culture medium. As depicted in Figure 7, when free DOX was administered to tumor cells, the red fluorescence of DOX exhibited a distinct signal in both acidic and normal environments, indicating direct permeation of DOX into tumor cells, leading to its abundance within tumor cells and posing a significant risk of damaging normal cells. This finding was consistent with the results of the cell viability experiment (Figure 6c). To demonstrate the role of CS conjugation in selective cellular internalization, intracellular fluorescence imaging of unconjugated PAMAM dendrimers and CS-conjugated PAMAM dendrimers was conducted through a comparative analysis. Compared to unconjugated PAMAM dendrimers, CS-conjugated PAMAM dendrimers exhibited improved fluorescence distribution in both acidic and normal environments. However, neither formulation surpassed the fluorescence signal of free DOX. These results suggest that CS-conjugated PAMAM dendrimers offer a more efficient pathway for cellular internalization and ensure regulated DOX release in response to acidity. An analysis of the underlying reasons for the increased cellular internalization of CS-conjugated PAMAM dendrimers may point to CS’s beneficial role in cell adhesive interactions.

**Figure 8:**
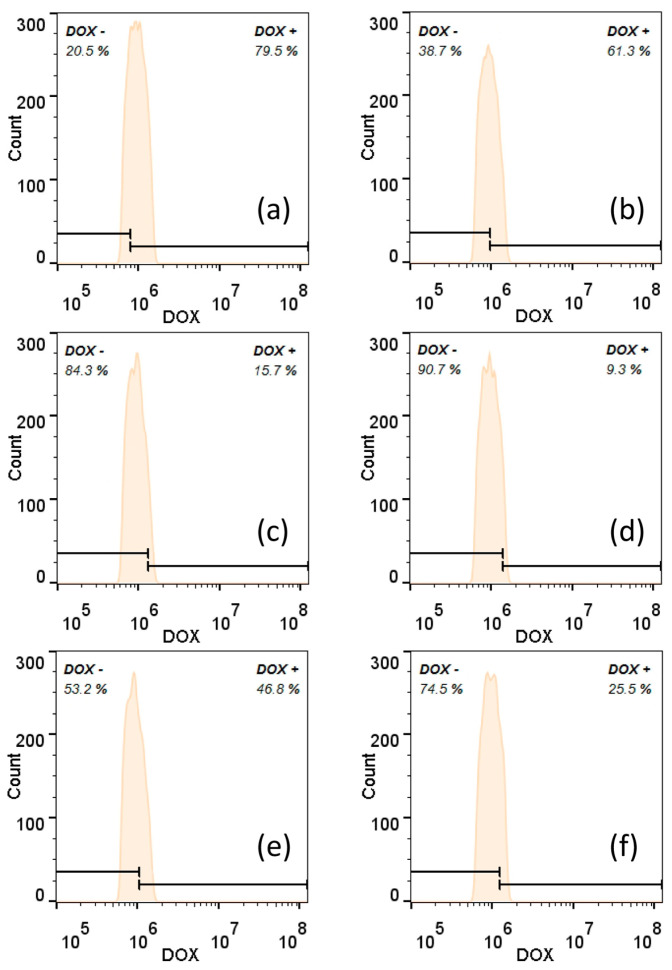
The representative flow cytometry profile of Skov 3 cells that were incubated with free DOX in an acidic environment (**a**) and a normal environment (**b**), DOX@PAMAM in an acidic environment (**c**) and a normal environment (**d**), and DOX@CS@PAMAM in an acidic environment (**e**) and a normal environment (**f**).

Correspondingly, flow cytometry was employed to gain quantitative insights into the internalization of nanoparticles in tumor cells (Figure 8, Figures S1 and S2). To validate the results obtained from intracellular fluorescence imaging, flow cytometry analysis was conducted on the free DOX group, unconjugated PAMAM dendrimer group, and CS-conjugated PAMAM dendrimer group in both acidic and normal environments in triplicate. Figure 8 presents the representative flow cytometry data, with its counterparts shown in Figure S1. As depicted in Figure 8, free DOX still exhibited the strongest fluorescence signal, with a mean fluorescence intensity (MFI) of 75.8 ± 13.7% in the acidic culture environment. Notably, the CS-conjugated PAMAM dendrimers showed significantly enhanced fluorescence intensity (52.7 ± 5.3%) when incubated with tumor cells in the acidic environment, nearly double the fluorescence intensity (24.3 ± 6.7%) observed in the normal environment. Conversely, the unconjugated PAMAM dendrimers displayed the weakest fluorescence intensity in both the acidic and normal environments. The heightened fluorescence intensity observed with CS-conjugated PAMAM dendrimers underscores the role of CS in facilitating the cellular internalization of this system.

Based on the above results, functionalized CS-conjugated PAMAM dendrimers demonstrated selective cellular uptake and increased antitumor efficacy. This enhancement is attributed to high-efficiency payloads provided by PAMAM, improved hydrophilicity through CS modification, and acid-responsive drug release via glutaraldehyde crosslinking. Specifically, compared to unconjugated PAMAM dendrimers, CS-conjugated PAMAM dendrimers showed significantly higher antitumor efficiency and drug distribution in tumor cells, enhancing cell affinity and tumor targeting. In an acidic environment, CS-conjugated PAMAM exhibited stronger growth inhibitory effects on tumor cells and controlled drug release due to the faster DOX release via acid-labile linking between CS and PAMAM. This pH-responsive release pattern also reduced cytotoxicity toward normal tissues. PAMAM dendrimers, characterized by their nanometer-scale topology and size, low water solubility, and low avidity toward tumor cells due to their material composition and structural characteristics, face challenges in clinical translation. In vitro experiments suggested that CS-conjugated PAMAM dendrimers possess superior antitumor activity compared to unconjugated PAMAM dendrimers. Numerous studies have demonstrated that CS modification can enhance the biological activities, mechanical properties, and physical properties of other drug carrier materials. For example, CS-modified mesoporous silica has been used to selectively target cancer cells and release DOX within them, achieving effective cancer treatment [27]. CS-modified hyaluronic acid has been utilized for its mucosal adhesiveness, targeting specific tissues [28]. CS-modified cholesterol-free liposomes have shown increased drug stability and permeability, thereby enhancing bioavailability [29]. Structural analyses, such as FTIR, TEM, and DLS, revealed that CS-conjugated PAMAM dendrimers are feasible and easily producible. These dendrimers enhance cellular internalization in tumor cells and exhibit pH-responsive release performance. Undoubtedly, CS modification compensates for the deficiencies of PAMAMs, such as weak hydrophilicity and low cell adhesion, and offers distinct advantages in controllable drug release. Further improvements in the CS/PAMAM conjugation ratio, hydrophobic–hydrophilic balance, and particle size could enhance their druggability and tumor targeting.

## 4. Conclusions

In conclusion, the proposed functional CS-conjugated PAMAM dendrimer underwent a thorough assessment of its physical and chemical properties, including confirmation of its assembled structure through FTIR, characterization of its dehydrated state morphology via TEM, and determination of its hydration state size using DLS. The DOX-loaded functional CS-conjugated PAMAM dendrimer exhibited outstanding acid-responsive properties attributed to the incorporated imine bond. Moreover, the unloaded functional CS-conjugated PAMAM dendrimer demonstrated good biocompatibility. The in vitro release experiment indicated that the drug release profile of the DOX@CS@PAMAM in an acidic environment was inferior to that of DOX@PAMAM in the same conditions. However, compared to the DOX-loaded unconjugated PAMAM dendrimer, the DOX-loaded functional CS-conjugated PAMAM dendrimer displayed an enhanced antitumor effect, showcasing improved tumor cellular internalization performance and regulated drug release behavior. Although not as fast and direct as free DOX, DOX@CS@PAMAM offers selective cellular uptake and controllable drug release. Consistent results from cell viability assays, fluorescence imaging, and flow cytometry underscored the significant enhancement in cellular internalization conferred by CS conjugation. The efficient CS conjugation achieved through glutaraldehyde crosslinking facilitated improved physical and chemical properties via chemical modification and nanotechnology. Overall, the synergistic combination of CS and PAMAM dendrimers represents a superior strategy for drug loading, delivery, and release, leading to selective cellular internalization.

## Data Availability

Data are available from the corresponding author with reasonable request.

## Supplementary Materials

The following supporting information can be downloaded at: https://www.mdpi.com/article/10.3390/s24154853/s1, Figure S1. Flow cytometry profile (counterpart 1) of Skov 3 cells that were incubated with free DOX in acidic environment (a) and normal environment (b), DOX@PAMAM in acidic environment (c) and normal environment (d) and DOX@CS@PAMAM in acidic environment (e) and normal environment (f). Figure S2. Flow cytometry profile (counterpart 2) of Skov 3 cells that were incubated with free DOX in acidic environment (a) and normal environment (b), DOX@PAMAM in acidic environment (c) and normal environment (d) and DOX@CS@PAMAM in acidic environment (e) and normal environment (f).
