# Supplementary material for: Selective Cellular Uptake and Druggability Efficacy through Functionalized Chitosan-Conjugated Polyamidoamine (PAMAM) Dendrimers
Source: Sensors (Basel). 2024 Jul 26;24(15):4853. doi: 10.3390/s24154853 (PMC11315009; doi:10.3390/s24154853)
Supplement: Supplementary file 1 [file sensors-24-04853-s001.zip › sensors-3054271-supplementary.pdf]

Selective Cellular Uptake and Druggability Efficacy Through Functionalized Chitosan-Conjugated Polyamidoamine (PAMAM) Dendrimers

Ye Hu [1], Jian Chen [2,*] and Wenyan Hu [1,*]

[1] Nanjing Institute for Food and Drug Control, Nanjing 211198, China; huye0210@163.com
[2] School of Chemistry and Chemical Engineering, Hunan University of Science and Technology, Xiangtan 411201, China
* Correspondence: chenjianpharm@hnust.edu.cn (J.C.); sbkxk@njifdc.org.cn (W.H.)

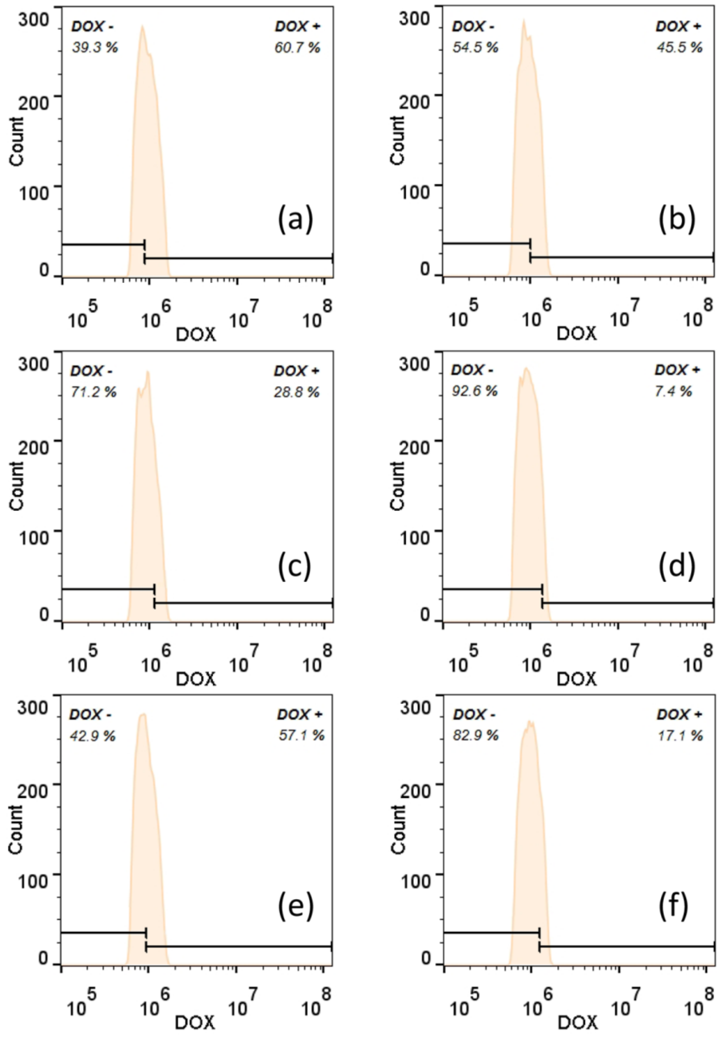

Figure S1. Flow cytometry profile (counterpart 1) of Skov 3 cells that were incubated with free DOX in acidic environment (**a**) and normal environment (**b**), DOX@PAMAM in acidic environment (**c**) and normal environment (**d**) and DOX@CS@PAMAM in acidic environment (**e**) and normal environment (**f**).

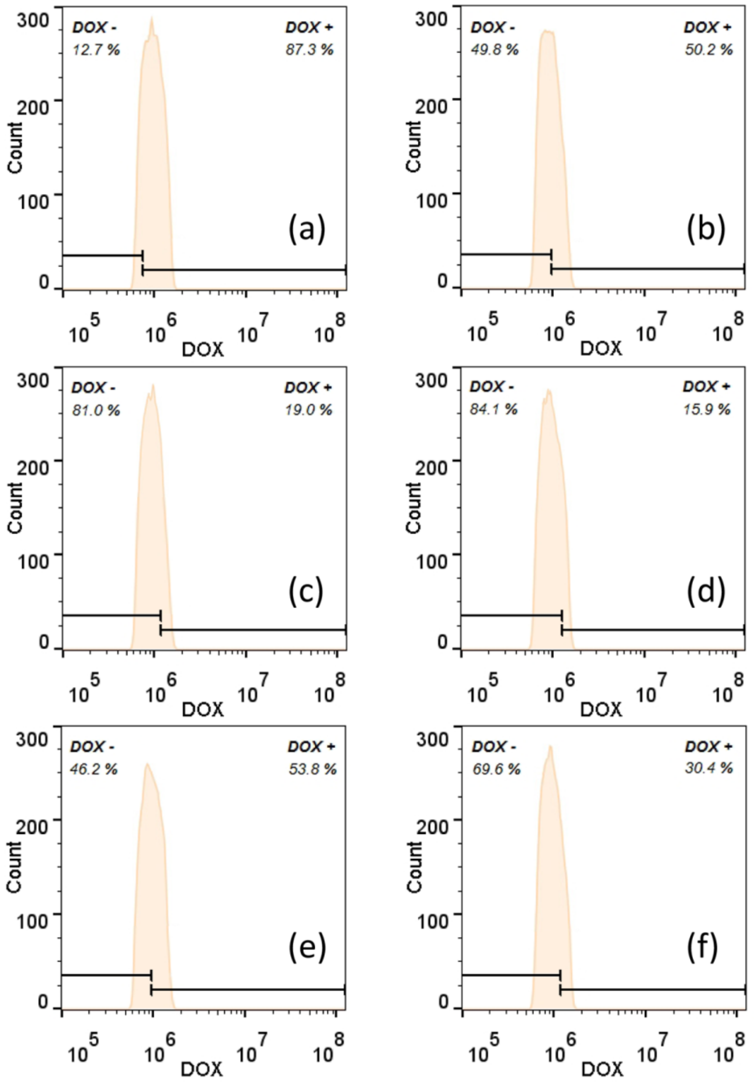

Figure S2. Flow cytometry profile (counterpart 2) of Skov 3 cells that were incubated with free DOX in acidic environment (**a**) and normal environment (**b**), DOX@PAMAM in acidic environment (**c**) and normal environment (**d**) and DOX@CS@PAMAM in acidic environment (**e**) and normal environment (**f**).
